# A fatal combination of situs inversus, pregnancy and cardiac arrest treated with an automated external defibrillator

**DOI:** 10.1007/s12471-016-0852-4

**Published:** 2016-05-23

**Authors:** S. Calle, M. De Leeuw, N. Mpotos, P. Calle, B. De Turck

**Affiliations:** 1 Faculty of Medicine and Health Sciences, Ghent University, Ghent, Belgium; 2 Forensic Pathology Department, Ghent University Hospital, Ghent, Belgium; 3 Department of Emergency Medicine, Sint Lukas General Hospital, Ghent, Belgium; 4 Faculty of Medicine and Health Sciences, University of Antwerp, Wilrijk, Belgium; 5 Department of Emergency Medicine, Maria Middelares General Hospital, Ghent, Belgium

## Answer

The AED delivered wrongful shocks, probably because the tachyarrhythmia with QRS complexes of low amplitude was considered to be ventricular fibrillation. Evidence for this comes from the ECG strips from the first AED rhythm analysis, clearly showing QRS complexes of a similar shape and amplitude, but at a lower rate. Furthermore, these small QRS complexes were continuously found from the start of the resuscitation attempt until the fourth shock (data not shown).

This case illustrates that the specificity of shock/no-shock decisions by the AEDs is not 100 % [1, 2]. Cardiologists should also be aware that AED sensitivity figures are only within a 90–95 % range, and that external artefacts during the analysis process (such as chest compressions) decrease the accuracy significantly. This implies that shock/no-shock decisions should be scrutinised before being used for diagnostic/therapeutic decision making and prognostication.

Remarkably, no cause of death could be detected on autopsy. Therefore, further research on the cardiac arrest risk stratification in dextrocardia in general, and during pregnancy in particular, is needed.

A third lesson concerns the low amplitude of QRS complexes detected by the AED electrodes placed in the conventional sternal-apical position [3]. Therefore, it seems reasonable in known dextrocardia cases to change to bi-axillary electrodes placement or a mirror-like approach (i. e. placement to the left of the sternum and in the right mid-axillary line). The reduced QRS amplitude in this dextrocardia case also argues against the use of the above-mentioned mirror-like approach in a standard cardiac arrest patient with a medical device implanted below the right clavicle.
